# Predicted spatial distribution of the primary Lassa virus reservoir, the multimammate mouse, in Africa

**DOI:** 10.3389/fpubh.2026.1916488

**Published:** 2026-10-13

**Authors:** Adeola Mariam Lateef, Marlon E. Cobos, Victor Ibukun Agbajelola, Ram K. Raghavan

**Affiliations:** 1 Department of Pathobiology and Integrative Biomedical Sciences, College of Veterinary Medicine, University of Missouri, Columbia, MO, United States; 2 Department of Ecology and Evolutionary Biology and Biodiversity Institute, University of Kansas, Lawrence, KS, United States; 3 Department of Public Health, College of Health Sciences, University of Missouri, Columbia, MO, United States; 4 MU Institute for Data Science and Informatics, University of Missouri, Columbia, MO, United States

**Keywords:** correlative modeling, ecological niche modelling, Lassa fever, *Mastomys natalensis*, one health

## Abstract

**Objective:**

This study predicts the potential spatial distribution of the multimammate mouse (*Mastomys natalensis*), the primary host of Lassa virus, a major public health concern across West Africa.

**Methods:**

Correlative maximum entropy models were constructed with occurrence records of *M. natalensis* from Global Biodiversity Information Facility, using long-term bioclimatic variables at ~1 km resolution as predictors. Candidate models were evaluated and best models were selected based on statistical significance, predictive ability, and model fitting and complexity. Models were projected to the African continent.

**Results:**

Median predictions from the best-supported models indicated extensive areas of moderate to high suitability across sub-Saharan Africa, with suitability concentrated within the tropical and subtropical belts of West, Central, and East Africa. Suitability declined sharply across arid regions, particularly the Sahara and parts of the Horn of Africa. Within Nigeria, a focal area of the study, the predicted suitability was broadly high across southern and central regions, with a gradual decline toward Sahel-adjacent northern areas. Sub-national projections for Lagos, Oyo, Edo, Kano, and the Federal Capital Territory revealed spatial heterogeneity in suitability, consistent with underlying climatic gradients in temperature and precipitation that shape habitat conditions for *M. natalensis*.

**Conclusion:**

These findings provide a spatial framework for prioritizing rodent surveillance and supporting targeted Lassa fever preparedness and control strategies.

## 1 Introduction

Lassa fever (LF) is a fatal viral hemorrhagic illness responsible for hundreds of deaths each year in West Africa. In severe cases of LF, multiple organs and tissues may be adversely affected, resulting in symptoms such as facial swelling, internal bleeding, severe anemia, low blood pressure, mental confusion, kidney failure, and, in extreme instances, coma and death within 14 to 20 days after symptoms begin (1). The causal agent, Lassa fever virus (LASV) (Family: Arenaviridae), is classified by the United States Centers for Disease Control and Prevention as a Category A bioterrorism agent. The disease is currently widely prevalent in West Africa; in Nigeria, where the disease is believed to have originated (2), 1,968 confirmed LF cases were reported between January 2022 and September 2023 (3). In 2025 alone, the Nigeria Centre for Disease Control and Prevention (NCDC) reported 1,171 suspected cases and 290 confirmed cases of Lassa fever, resulting in 53 deaths (4, 5).

Lassa fever has traditionally been described as having two primary endemic zones in West Africa, one focused around Sierra Leone and Liberia, and the other in Nigeria (6). However, in recent years, other countries in the region have begun reporting regular or sporadic cases (7, 8). Historically, Lassa fever was concentrated primarily in Nigeria and the Mano River Union countries of Sierra Leone, Liberia, and Guinea. However, evidence supports a broader recognized endemic range, with outbreaks and sporadic cases documented in other West African countries, including Benin, Togo, Ghana, Mali, Burkina Faso, and Côte d’Ivoire (9, 10). Lassa fever is a rodent-borne disease, and its geographic distribution and endemicity can be expected to be influenced in turn to some extent by the distribution of its reservoirs. The primary reservoir host transmitting this virus to humans is rodents of the *Mastomys* genus. Transmission occurs through contact with their urine, feces, saliva, or blood (11). Beyond the *Mastomys* species, other rodents such as *Hylomyscus pamfi*, *Mus musculus* (house mouse), and *Rattus rattus* (roof rat) have also been implicated in spreading LASV; however, their role in the epidemiology of this disease is considered to be relatively minor compared to the multimammate mouse, *Mastomys natalensis* (Family: Muridae), considered to be the main host for LASV (12, 13).

The multimammate mice are wild rodents commonly found living alongside humans in Africa and are the most common rodent species in sub-Saharan Africa. Prior modeling work suggests that roughly 37.7 million people residing in 14 nations across sub-Saharan Africa, particularly within the West African sub-region, are exposed to potential Lassa virus transmission due to the presence of established rodent reservoirs (14). In addition to being the main reservoir of LASV, they are known to host a wide variety of other pathogens (15), such as papillomavirus, herpesviruses, polyomavirus, *Yersinia* spp., *Borrelia* spp., and the parasite *Leishmania* spp., (16). In African agriculture, *M. natalensis* is also regarded as the leading rodent pest, with population outbreaks capable of causing crop losses of up to 80% at both local and regional scales (17). Understanding the spatial extent, distribution patterns, and variation in distribution density of *M. natalensis* is therefore central to elucidating the epidemiology and disease ecology of Lassa fever (LF). Because this rodent frequently occurs in and around human settlements, its local abundance is strongly influenced by anthropogenic factors such as agriculture, food storage practices, and settlement structure. However, at broader geographic scales, the distribution of wild rodent populations is largely constrained by environmental conditions that determine habitat suitability. This ecological relationship provides the basis for correlative species distribution modeling approaches, which integrate known occurrence records with environmental predictors to estimate the spatial extent and distributional patterns (18).

Using this framework, we modeled the potential spatial distribution of *M. natalensis* in Africa. Although previous studies have examined the geographic distribution of Lassa fever and its principal reservoir using ecological and climatic modeling approaches (14), our study provides an updated, reservoir-focused assessment using contemporary occurrence records and a more rigorous ecological niche modeling framework. We conducted the analysis at two scales: at the continental African scale to elucidate the broader distributional envelope of the species, and at the scale of Nigeria, which consistently reports the highest burden of LF infections. The estimates of environmentally suitable areas provide an important approximation of the species’ potential distribution and can help address key knowledge gaps in LF epidemiology by identifying regions where host populations are most likely to occur and sustain transmission risk.

## 2 Materials and methods

### 2.1 Occurrence data

Occurrence records of *M. natalensis* were obtained from the Global Biodiversity Information Facility (19), resulting in 20,631 records collected between 1950 and 2023. These records contained geographic coordinates indicating locations where the species had been observed. However, the raw dataset exhibited issues such as duplicate entries, missing geographic coordinates, and regional overrepresentation. To improve data quality, several filtering and cleaning steps were applied. First, records lacking geographic coordinates were removed. The coordinates were then visualized in ArcGIS, revealing errors including mislocated latitude and longitude points. Following this inspection, only valid geographic points were retained, and erroneous entries, such as duplicates and location centroids, were removed following procedures like those implemented in the CoordinateCleaner package (20) in R (21). To further refine spatial accuracy, occurrence points falling on invalid environmental raster cells (i.e., cells with missing or NoData environmental values) but located within 5 km of a valid cell containing environmental data for the predictor layers were moved to the nearest valid cell using *kuenm2* (22). This procedure was applied to retain records located close to the boundary of valid environmental coverage while limiting coordinate displacement to a maximum of 5 km; records without a valid raster cell within this distance were excluded. Duplicate points within the same raster cell were also removed using kuenm2 to maintain one occurrence per environmental pixel. To ensure ecological relevance and geographic accuracy, only occurrence records from sub-Saharan Africa, the native range of *M. natalensis*, were retained (Figure 1A). This step was essential for avoiding inclusion of non-native or erroneously placed occurrences. Additional cleaning was performed in R to eliminate remaining zero-coordinate values. Occurrence records of *M. natalensis* in Nigeria were georeferenced and visualized in ArcGIS to illustrate the species’ spatial distribution (Figure 1B).

**Figure 1:**
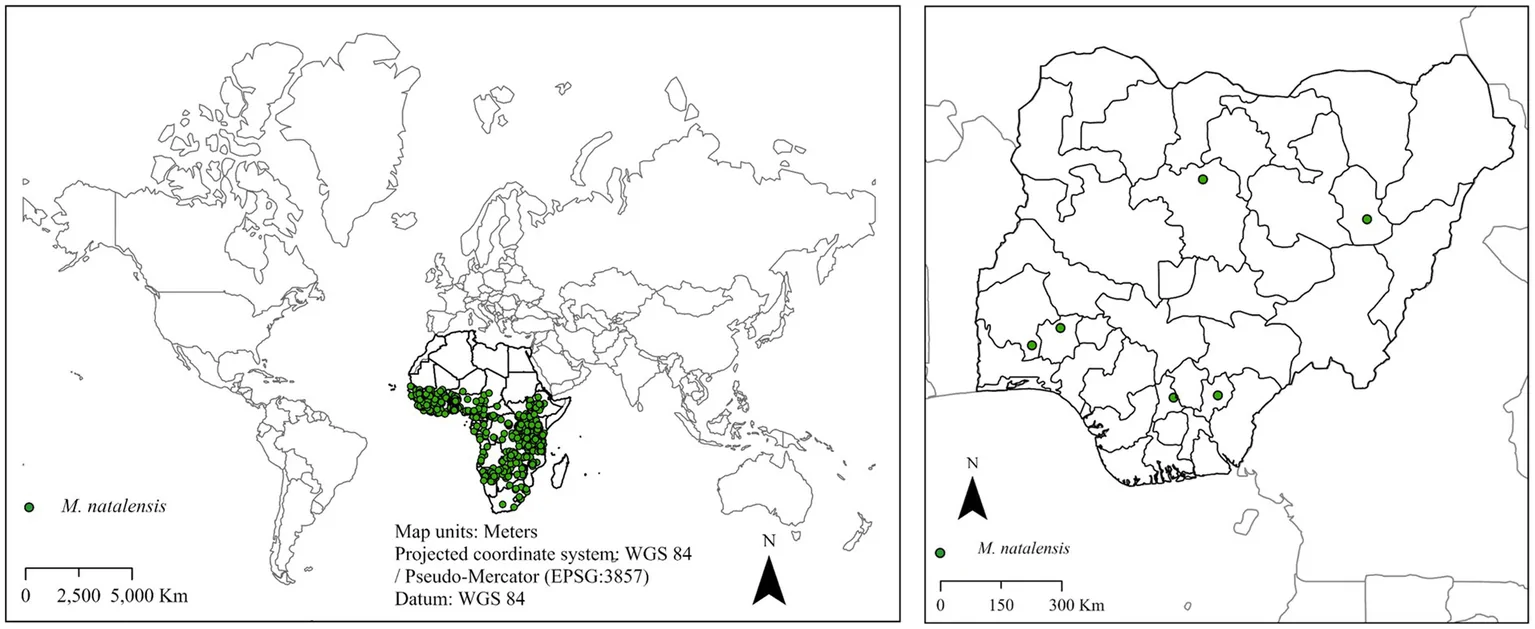
Geographic distribution records of *M. natalensis* in Africa (Left) and Nigeria (right). Green dots represent occurrence records obtained from (GBIF).

### 2.2 Environmental data preparation

We obtained 19 bioclimatic variables from WorldClim version 2 (23), derived from monthly temperature and precipitation averages and representing biologically meaningful dimensions of climate variability. These environmental predictor layers were downloaded at a 0.5 arc-minute (~1 km) spatial resolution using the geodata package and clipped to the African continent for computational efficiency and ecological focus. The layers were masked further based on an area relevant for model calibration and used to explore patterns of spatial autocorrelation and for ecological niche modeling (see below).

### 2.3 Pre-modeling steps and ecological niche modeling

To mitigate biases deriving from spatial autocorrelation, spatial thinning was applied using the *spThin* package (24). Thinned datasets were generated at three spatial distances: 20 km, 30 km, and 50 km (Supplementary Table S1). To examine the effect of thinning on spatial autocorrelation, Moran’s I was calculated using the *moranfast* package version 1.0 (25) for four representative bioclimatic variables: BIO1 (Annual Mean Temperature), BIO7 (Temperature Annual Range), BIO12 (Annual Precipitation), and BIO15 (Precipitation Seasonality). For each variable, environmental values extracted at the occurrence locations were analyzed together with the longitude and latitude coordinates of the corresponding records using the moranfast function. Moran’s I was calculated separately for the unthinned dataset and for datasets thinned at 20, 30, and 50 km to evaluate changes in the magnitude and statistical significance of spatial autocorrelation following thinning. These four variables were used specifically for the Moran’s I analysis, whereas all 19 bioclimatic variables were included in the subsequent PCA used for model calibration.

We employed ecological niche modeling to estimate the potential distribution of *M. natalensis* across sub-Saharan Africa using a correlative approach. The modeling followed established ENM protocols (26, 27), utilizing maxnet (16) through the package kuenm2 (22). We restricted predictors to climatic variables because the objective was to characterize broad-scale climatic suitability for *M. natalensis* across its extensive African range rather than to model local habitat occupancy. Temperature and precipitation represent broad-scale abiotic constraints that can influence the species’ distribution and population dynamics, although non-climatic factors such as vegetation, land use, food availability, biotic interactions, and human-modified habitats may influence its realized distribution at finer spatial scales. Accordingly, the resulting predictions are interpreted as areas of relative climatic suitability rather than the complete realized distribution or local abundance of *M. natalensis*.

To define an accessible area for the species (M), we followed the BAM framework (28), which conceptualizes species distributions as the intersection of biotic interactions (B), abiotic suitability (A), and dispersal accessibility (M). The initial M region was delineated to encompass sub-Saharan Africa, reflecting the known native range of *M. natalensis* and realistic broad-scale dispersal limits. Because accessibility is not static and may reflect historical and ecological processes, we further refined M using dispersal-based simulations rather than assuming a fixed geographic boundary.

We implemented dispersal simulations using the R package *grinnell* (29) which generates accessible areas based on repeated dispersal events from occurrence locations under specified ecological assumptions (30). Simulations were conducted using the 30-km spatially thinned occurrence dataset to reduce spatial autocorrelation while retaining representative coverage of the species’ range. A grid of dispersal parameter combinations was explored, including kernel spread values of 0.5, 1, and 2 and dispersal event counts of 125, 250, and 500, producing multiple candidate accessible-area (M) surfaces. The final calibration area was based on the simulation using a kernel spread value of 2 and 500 dispersal events, representing a broader dispersal scenario for *M. natalensis*.

Environmental predictor variables and occurrence data were prepared for ENM using kuenm2. To reduce multicollinearity among predictors and summarize major environmental gradients, Principal Component Analysis (PCA) was performed on all 19 WorldClim bioclimatic variables using environmental values extracted from occurrence and background locations. Predictor variables were centered and scaled prior to PCA to account for differences in measurement units and magnitude. Principal components were retained using the criteria implemented in kuenm2, with a target cumulative explained variance of 95%, with components explaining ≤5% of the variance individually excluded. The retained principal components were subsequently used as environmental predictors during ecological niche model calibration.

Model calibration was performed and candidate models were generated using combinations of at least two retained principal components and five feature class configurations: quadratic (Q), linear quadratic (LQ), linear product (LP), quadratic product (QP), and linear quadratic product (LQP). These configurations were evaluated across nine regularization multipliers (0.1, 0.25, 0.5, 0.75, 1, 2, 3, 4, and 5), yielding 495 candidate model configurations. Model performance was assessed via a data cross-validation with 5 k-fold partitions, using three criteria: (i) statistical significance via partial Receiver Operating Characteristic (ROC) analysis (31), (ii) omission rate at a 5% omission threshold (32), and (iii) model complexity measured by the corrected Akaike Information Criterion (AICc) following Warren & Seifert (33). Based on these criteria, only best- performing models, balancing predictive power and complexity, were retained for final modeling.

The best models were then refitted with 10 k-fold replicates to account for variability, and these final models were saved for further analysis. Final models, as well as their projections across different times or regions, were developed using parameters chosen during the calibration phase. These models were generated using unrestricted extrapolation, allowing predictions beyond the environmental conditions represented in the calibration data (27). Variable contributions were evaluated, and ecological response curves were generated for the environmental variables included in models. Consensus projections were generated by summarizing predictions across all selected models. The median consensus map was used as the primary output because it represents the central tendency of habitat suitability predictions while reducing the influence of extreme values among individual model replicates. Standard deviation across model replicates was used to represent variability in predicted suitability. In addition, Mobility-Oriented Parity (MOP) analysis was used to identify areas with environmental conditions non-analogous to those represented during model calibration and to highlight areas where predictions involved environmental extrapolation.

## 3 Results

### 3.1 Occurrence data processing and spatial distribution

A total of 20,631 occurrence records for *M. natalensis* were obtained from GBIF and removal of invalid coordinates or implausible geographic coordinates reduced the data to 16,896 records. Restricting the dataset to the native range of sub-Saharan Africa (latitude −35 to 20; longitude −20 to 55) resulted in 16,894 records following exclusion of 2 non-native occurrences. Duplicate geographic coordinates were removed, reducing the dataset to 982 unique records, and an additional record with zero coordinates was excluded, yielding 981 occurrences for further processing.

Alignment of occurrence records with environmental raster layers retained 979 correctly positioned points. To ensure one occurrence per grid cell, raster-based deduplication reduced the dataset to 950 records. Spatial thinning at minimum distances of 20, 30, and 50 km further reduced the dataset to 588, 522, and 405 records, respectively (Supplementary Table S1). Moran’s I analysis showed that thinning reduced the magnitude of spatial autocorrelation across the four evaluated environmental variables (BIO1, BIO7, BIO12, and BIO15), although significant spatial structure remained after thinning (*p* < 0.001; Supplementary Table S2). For example, Moran’s I for BIO1 decreased from 2.10 in the unthinned dataset to 0.37, 0.35, and 0.32 at 20, 30, and 50 km, respectively, while BIO15 decreased from 0.80 to 0.35, 0.32, and 0.28. The 30-km thinned dataset (*n* = 522) was selected as an intermediate thinning distance that reduced spatial dependence while retaining sufficient occurrence records and geographic representation across the species’ range. This dataset was subsequently used for accessible-area (M) simulations and ecological niche model calibration.

The resulting records indicate that *M. natalensis* is widely distributed across sub-Saharan Africa, with high record density in West, Central, and East Africa. In West Africa, notable clusters occur in Nigeria, Sierra Leone, and Guinea, overlapping regions historically associated with Lassa fever endemicity. Within Nigeria, occurrences are widespread but spatially clustered, with the highest concentrations in the southwestern states (Oyo, Ogun, Osun, Ondo, and Lagos), as well as in southern and north-central regions. In contrast, relatively sparse records are observed in the far northwest and parts of the northeast.

### 3.2 Model calibration and selection

Examination of the PCA loadings shows that PC1 was mainly associated with annual precipitation (bio12), temperature seasonality (bio4), annual temperature range (bio7), and precipitation seasonality (bio15). PC3 was primarily related to precipitation extremes and seasonal rainfall variation (BIO12 and BIO15). PCA identified four principal components (PC1–PC4) that individually explained more than 5% of the total variance, accounting for 42.77%, 30.34%, 10.26%, and 5.87%, respectively, and 89.23% cumulatively. During model selection, the best-performing models retained PC1–PC3 as environmental predictors; together, these three components explained 83.36% of the total environmental variance (Tables S3–S4).

A total of 495 candidate models were evaluated during calibration. All models converged successfully, and none were excluded due to fitting failure. Based on the omission rate criterion (≤ 5%), 316 models were retained, indicating that a substantial proportion of candidate models adequately predicted known occurrences. From this subset, three models met the ΔAICc <2 criterion and were selected as the top-performing model under an information-theoretic framework (Table 1) (32). The selected models also produced significant partial ROC values, confirming performance significantly better than random expectations.

**Table 1:** Summary of candidate ecological niche models for *Mastomys natalensis.*

Model characteristic	Model 452	Model 458	Model 459
Model formula	PC1 + PC2 + PC3 + I(PC1^2^) + I(PC2^2^) + I(PC3^2^) + PC1: PC2 + PC1: PC3 + PC2: PC3–1	PC1 + PC2 + PC3 + I(PC1^2^) + I(PC2^2^) + I(PC3^2^) + PC1: PC2 + PC1: PC3 + PC2: PC3–1	PC1 + PC2 + PC3 + I(PC1^2^) + I(PC2^2^) + I(PC3^2^) + PC1: PC2 + PC1: PC3 + PC2: PC3–1
Regularization multiplier	2.00	0.10	0.25
Feature class	LQP	LQP	LQP
Omission rate	0.046	0.043	0.045
Mean AUC ratio	1.0626	1.0677	1.0659
Partial ROC *p*-value	<0.001	<0.001	<0.001
AICc	7521.206	7523.004	7523.105
No. of parameters	5	8	8
ΔAICc	0.000	1.798	1.899

### 3.3 Variable importance and response curves

The contributions of PCs to models showed that PC1 was the dominant predictor, contributing approximately 68–70% of the total variation explained by the final models (Figure 2a). In comparison, PC2 and PC3 contributed less, each accounting for 15–17% of the overall variation. The narrow spread observed across the boxplots indicates consistent contributions of the principal components among selected models.

**Figure 2:**
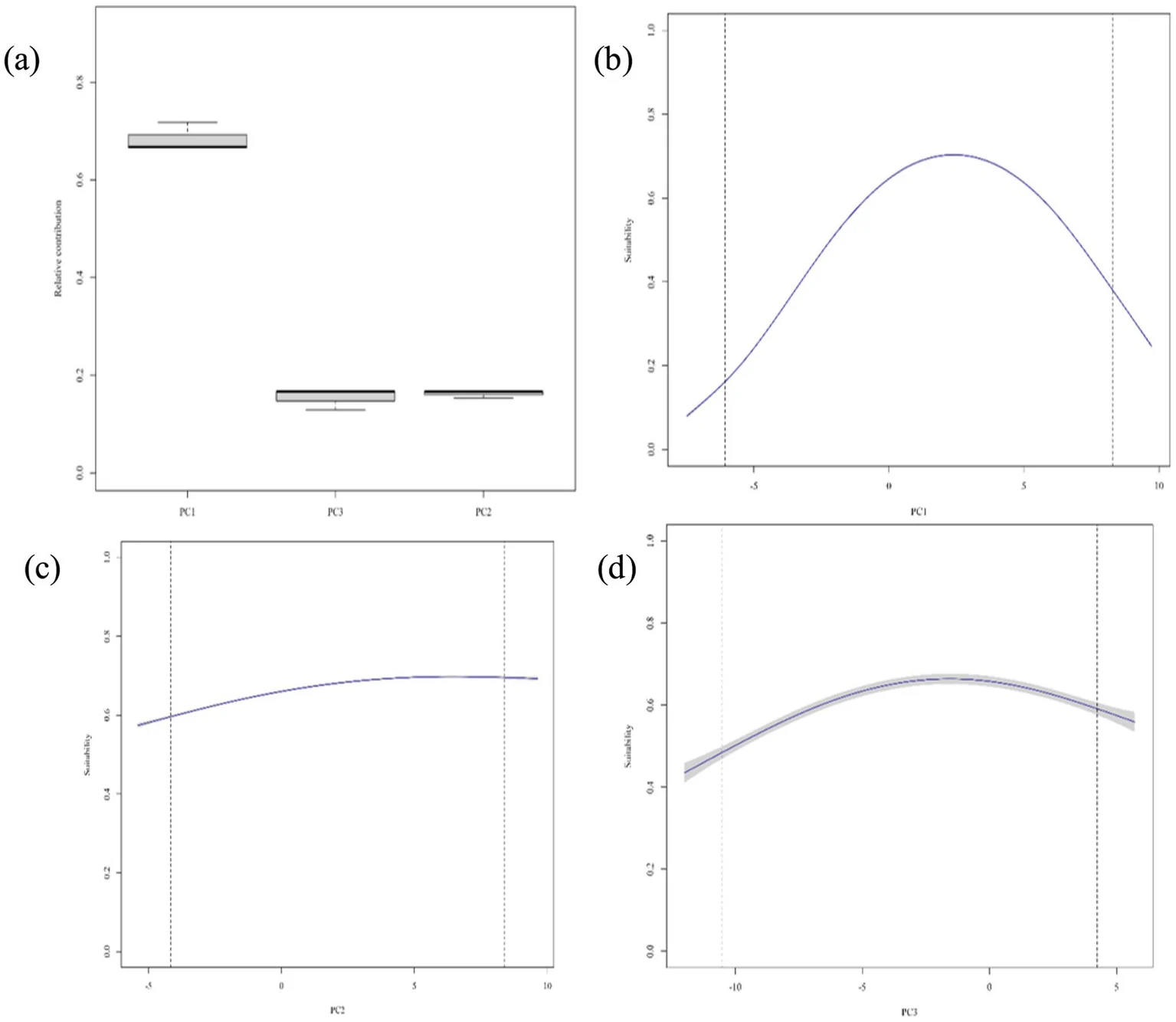
**(a)** Relative contribution of environmental predictors derived from principal component analysis to the final ecological niche model for *Mastomys natalensis*. Response curves for **(b)** PC1, **(c)** PC2, and **(d)** PC3 showing relationships between environmental gradients and predicted habitat suitability. Suitability exhibited unimodal responses to PC1 and PC3, peaking at intermediate environmental conditions, while PC2 showed a slight positive trend. Dashed vertical lines represent the environmental limits used for model calibration.

Response curve analyses for PCs 1–3 (Figures 2b–d) revealed distinct relationships with predicted suitability. PC1 exhibited a pronounced unimodal response, with suitability increasing toward intermediate values and declining at higher values, indicating preference for moderate conditions along this dominant climatic gradient. PC2 showed a generally positive trend, with suitability gradually increasing and stabilizing at higher values. PC3 also displayed a unimodal response, but with a broader, more moderate peak, suggesting relatively wider tolerance to the environmental conditions represented by this component.

### 3.4 Predicted suitability across Africa and Nigeria

At the continental scale, the median prediction map (Figure 3a) indicated extensive areas of moderate to high suitability across sub-Saharan Africa. High suitability was concentrated within the tropical belt, including West Africa, Central Africa, East Africa, and parts of southern Africa. Notable areas of elevated suitability were observed across the Guinea savanna and forest–savanna mosaic of West Africa, the Congo Basin region, the East African highlands and adjacent lowlands, and parts of southeastern Africa, including Madagascar. In contrast, suitability was consistently low across arid and semi-arid regions, particularly the Sahara Desert and portions of the Horn of Africa, as well as in southernmost areas associated with cooler climatic conditions and distinct biomes.

**Figure 3:**
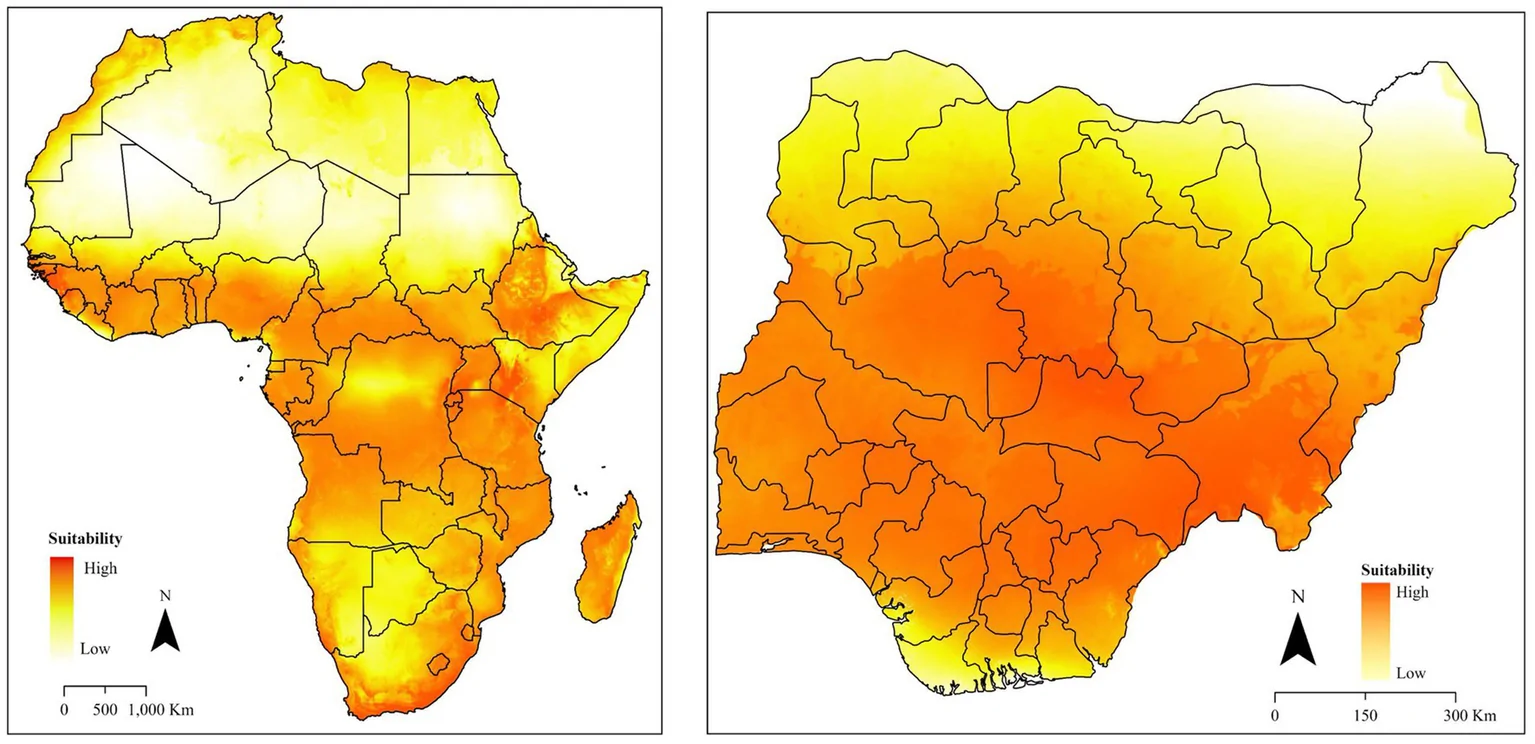
Median suitability for *M. natalensis* in Africa **(left)** and in Nigeria **(right)**, warmer colors (orange–red) indicate higher suitability, while lighter colors (yellow–white) represent lower suitability.

Within Nigeria, the median prediction indicated broadly suitable conditions across much of the country (Figure 3b). Suitability was highest across the southern and central regions, including the South-West, South–South, South-East, and extending into the North-Central zone. Suitability declined toward the far north, particularly in Sahel-adjacent areas where climatic constraints likely limit persistence.

State-level projections revealed notable sub-national variation in predicted habitat suitability across Nigeria (Figures 4a–e). In Oyo State, suitability was generally moderate to high, with elevated suitability concentrated in the eastern and southeastern regions, while lower suitability occurred in parts of the western and central areas (Figure 4a). Edo State exhibited consistently high suitability across much of the state, particularly in the northern and eastern regions, with comparatively lower suitability toward the southern margin (Figure 4b).

**Figure 4:**
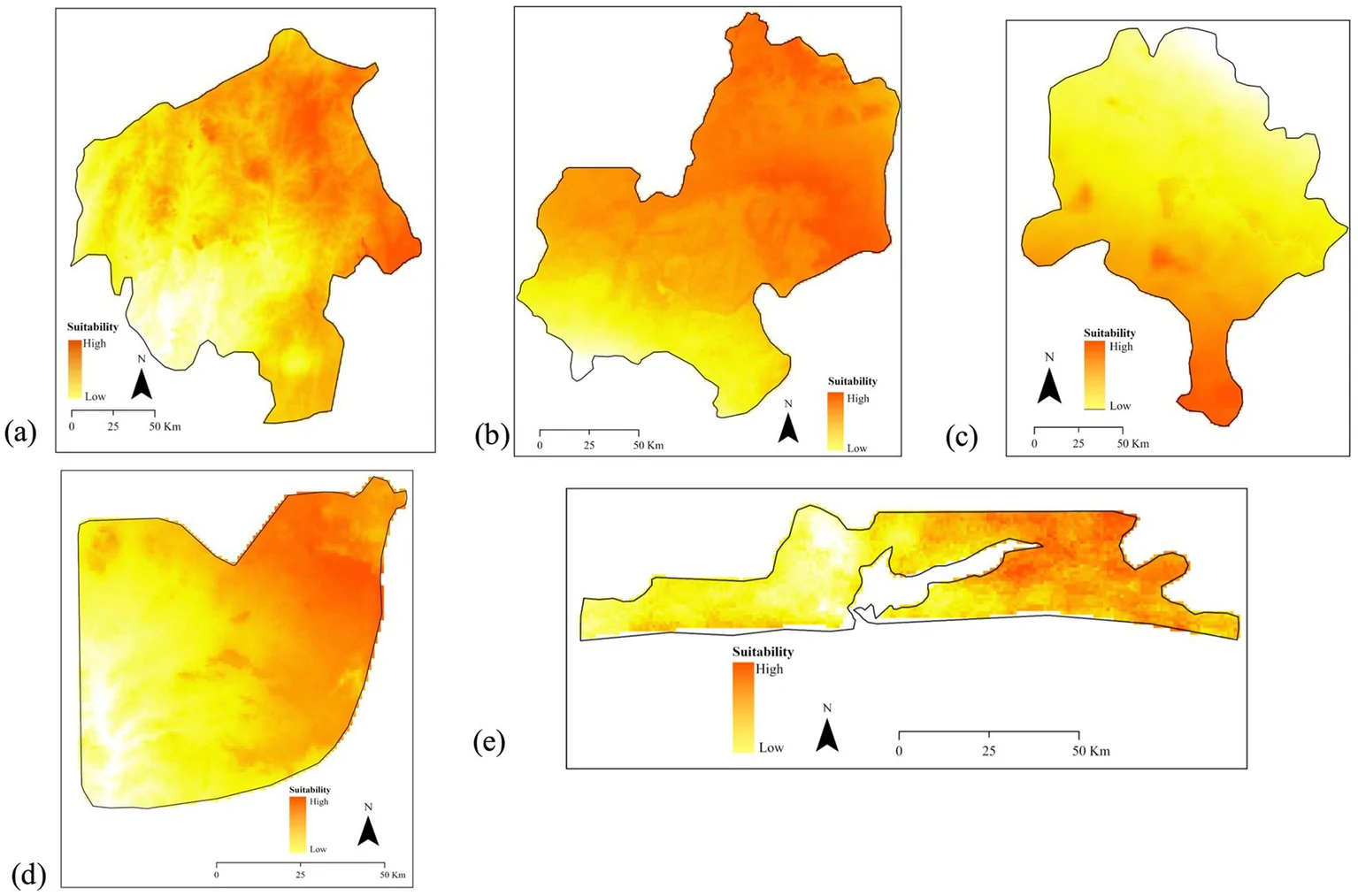
Median habitat suitability prediction for *M. natalensis* Oyo State **(a)**, Edo state **(b)**, Kano state **(c)**, Abuja **(d)**, and Lagos state **(e)**, Nigeria.

In Kano State, suitability was predominantly low to moderate, although localized areas of higher suitability were observed in the southern and southwestern portions of the state (Figure 4c). The Federal Capital Territory (FCT) displayed moderate to high suitability throughout most areas, with the highest suitability concentrated toward the northern and northeastern sections (Figure 4d). Lagos State showed a clear west–east suitability gradient, with lower suitability in the western region and progressively higher suitability toward the eastern and coastal peri-urban areas (Figure 4e). Overall, these state-level patterns demonstrate considerable spatial heterogeneity in habitat suitability at finer geographic scales.

Variability among model predictions, measured using the standard deviation across model replicates, showed distinct spatial patterns (Figure 5). Across Africa, variability was generally low across much of West and Central Africa, with localized areas of higher variability in coastal West Africa, East Africa, the Horn of Africa, and southern Africa. Within Nigeria, variability was generally low across northern and central regions but increased toward the southern coast, particularly around the Niger Delta.

**Figure 5:**
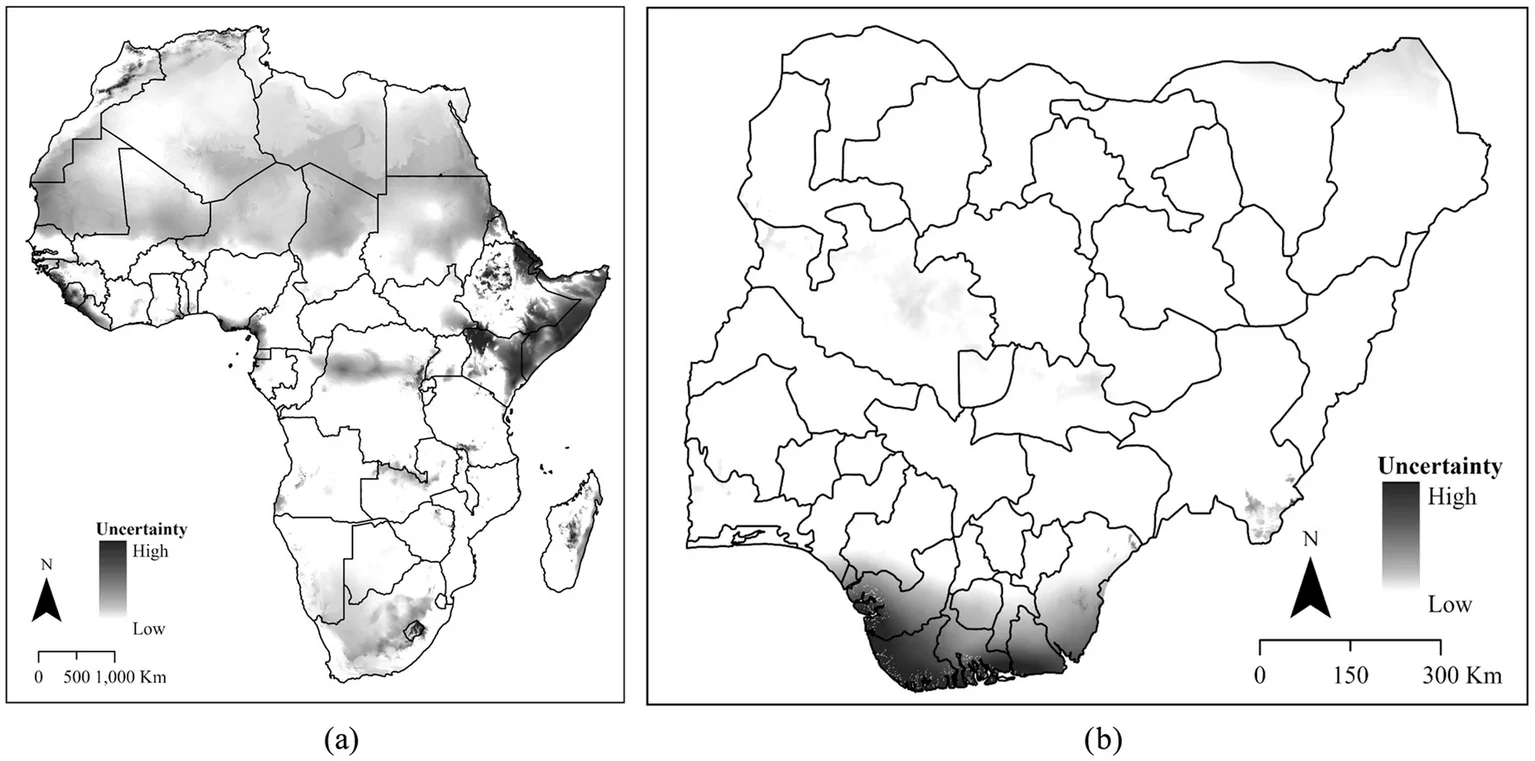
Uncertainty in predicted suitability for *M. natalensis* in Africa **(a)** and Nigeria **(b)**. Darker areas indicate higher uncertainty, while lighter areas represent lower uncertainty.

MOP analysis showed that 90.75% of evaluated raster cells across Africa were environmentally analogous to calibration conditions, whereas 9.25% contained at least one principal component outside its calibration range (Figure 6). Most extrapolation involved only one PC (9.17% of all cells), while extrapolation involving two (0.08%) or three PCs (<0.01%) was rare. Low-end extrapolation was predominantly associated with PC1, whereas high-end extrapolation was more spatially restricted and primarily associated with PC2 and PC3. Predictions within these environmentally non-analogous areas should therefore be interpreted cautiously because they involve conditions outside those represented during model calibration.

**Figure 6:**
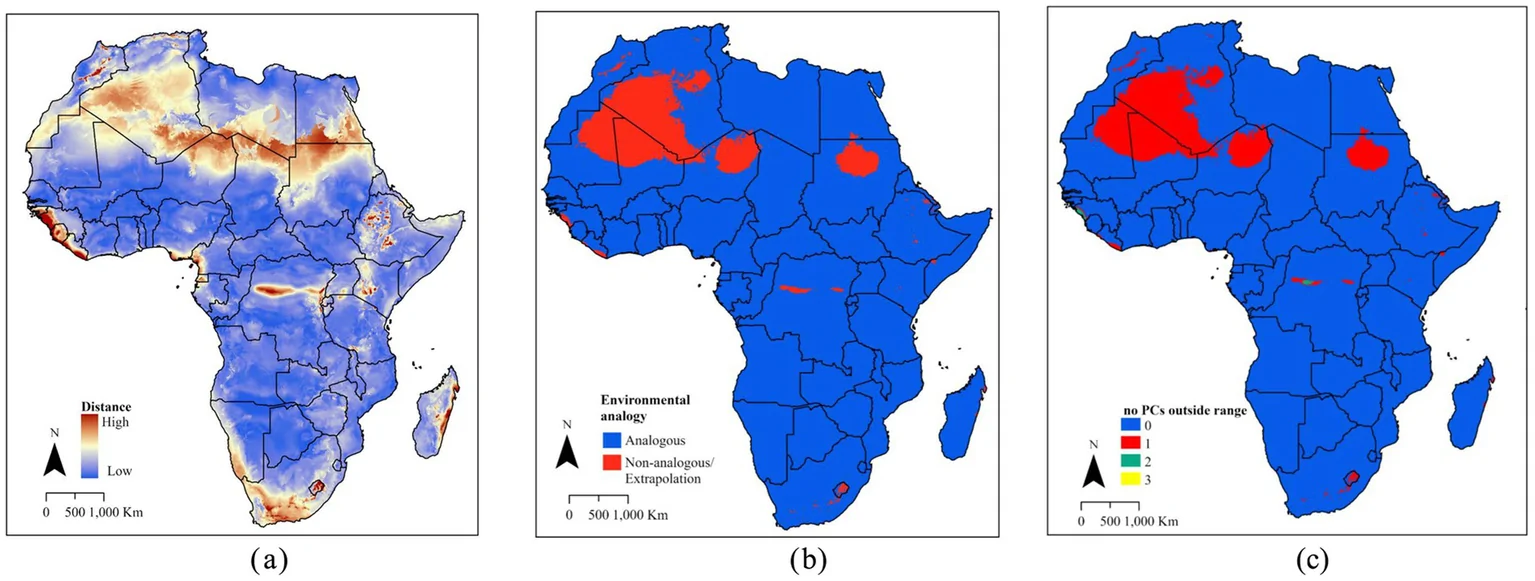
Environmental extrapolation risk assessed using Mobility-Oriented Parity (MOP) analysis across Africa. **(a)** Environmental distance from conditions represented in the model calibration area; **(b)** distribution of environmentally analogous and non-analogous conditions, with non-analogous areas indicating extrapolation; and **(c)** number of principal components (PCs) outside their calibration ranges.

## 4 Discussion

Our results demonstrate broad climatic suitability for *M. natalensis* across sub-Saharan Africa, extending considerably beyond the currently recognized geographic distribution of Lassa virus (LASV). At the continental scale, median habitat suitability was concentrated across tropical and subtropical regions of West, Central, and East Africa, with particularly high suitability in Nigeria, Guinea, Sierra Leone, Ghana, Cameroon, and the Democratic Republic of Congo, extending into parts of Ethiopia, Uganda, Kenya, Tanzania, southeastern Africa, and Madagascar (Figure 4a). Although relatively high suitability was predicted in parts of Madagascar, Madagascar was outside the accessible area (M) used for model calibration and high suitability should not be interpreted directly as presence. These patterns align with the species’ documented preference for savanna ecosystems, forest–savanna mosaics, and human-modified agricultural landscapes characterized by warm temperatures and moderate rainfall (34).

In West Africa, predicted suitability hotspots overlapped extensively with regions historically associated with Lassa fever transmission (35, 36). However, the model also identified environmentally suitable regions in Central, East, and southern Africa where reported Lassa fever occurrence remains limited. The broad geographic distribution of *M. natalensis* across sub-Saharan Africa contrasts with the more restricted occurrence of LASV, indicating that the presence of the principal reservoir alone is insufficient to explain the geographic distribution of the virus and should not be interpreted as Lassa fever risk. Several processes may contribute to this spatial mismatch, including phylogeographic structure among *M. natalensis* populations, geographic barriers to host and virus dispersal, variation in reservoir competence, and associations between particular rodent and arenavirus lineages (35, 36). Consequently, suitable areas predicted in Central, East, and southern Africa may provide climatic conditions favorable for *M. natalensis* without necessarily supporting LASV circulation. Understanding why LASV remains geographically constrained relative to its broadly distributed principal reservoir therefore represents an important area for further ecological and epidemiological investigation.

Within Nigeria, moderate to high climatic suitability for *M. natalensis* was widespread across much of southern and central Nigeria (Figure 3b). These areas are characterized by climatic conditions that appear favorable for the species, whereas suitability generally declined toward the far north, particularly in Sahel-adjacent zones characterized by lower rainfall and greater temperature variability. In Kano State, suitability was predominantly low to moderate, with localized areas of higher suitability in the southern and southwestern portions of the state.

State-level provided a closer view spatial heterogeneity of habitat suitability across Oyo, Edo, Lagos, Kano, and the Federal Capital Territory (Figures 4a–e). Oyo, Edo, and Lagos generally showed moderate to high climatic suitability, whereas Kano exhibited predominantly lower suitability with localized areas of higher suitability. These state-level patterns represent geographic subsets of the continental climatic suitability surface and should not be interpreted as effects of population density, land use, urbanization, or other local factors that were not included as predictors in the model.

The predicted distribution of *Mastomys natalensis* across Africa was strongly influenced by climatic conditions, particularly temperature and precipitation patterns. These environmental factors likely shape habitat suitability through their effects on resource availability, reproduction, and survival, consistent with previous studies of African rodent ecology (37). The variability maps showed that model predictions were relatively consistent across much of the study area, although distinct geographic areas exhibited greater variability (Figure 5). In Nigeria, the concentration of higher variability along the southern coastal region indicates greater variation among model predictions in these areas and suggests that suitability estimates there should be interpreted with greater caution. Similarly, localized areas of elevated variation were observed across parts of East and southern Africa. These patterns emphasize the importance of considering prediction variability alongside the median suitability surface rather than interpreting suitability estimates alone. However, the standard deviation represents variability among model predictions and does not account for other sources of uncertainty, including sampling bias, environmental novelty, or extrapolation beyond the calibration area.

MOP analysis indicated that environmental extrapolation was limited to a relatively small proportion of the projection area and was concentrated primarily in northern Africa. Predictions within these non-analogous areas should therefore be interpreted cautiously because they involve environmental conditions outside those represented during model calibration, although environmental novelty does not necessarily imply that the predictions are incorrect.

Collectively, the findings of this study provide a spatially explicit ecological framework for improving Lassa fever surveillance, preparedness, and targeted intervention strategies in Nigeria and across Africa. Although disease incidence was not modeled directly, identifying areas of high climatic suitability for *M. natalensis* can help prioritize locations for targeted rodent surveillance and further field investigation. These suitability estimates should not be interpreted as direct measures of rodent abundance, Lassa virus presence, or human spillover risk. Assessing spillover risk would require integrating reservoir suitability with additional information on rodent abundance and infection status, human–rodent contact, and epidemiological surveillance data.

Model performance was evaluated using cross-validation approaches which represent internal evaluation procedures. As we made concerted efforts to reduce spatial-autocorrelation biases, we are confident of the reliability of our evaluation methods. Future studies in the field could generate new occurrence independent data that can be used to evaluate model predictions more robustly.

## Data Availability

The original contributions presented in the study are included in the article/Supplementary material, further inquiries can be directed to the corresponding author.
