# Supplementary material for: Predicted spatial distribution of the primary Lassa virus reservoir, the multimammate mouse, in Africa
Source: Front Public Health. 2026 Oct 13;14:1916488. doi: 10.3389/fpubh.2026.1916488 (PMC13710268; doi:10.3389/fpubh.2026.1916488)
Supplement: Supplementary file 1 [file Table_1.docx]

**Supplementary data**

**Table S1**. Summary of occurrence records retained following spatial thinning at different thinning distances for *Mastomys natalensis*.

| Thinning Distance | Record Counts Obtained | Maximum Records After Thinning |
| --- | --- | --- |
| 20 km | 586, 587, 588 | 588 |
| 30 km | 520, 521, 522 | 522 |
| 50 km | 404, 405 | 405 |
