# Supplementary material for: Predicted spatial distribution of the primary Lassa virus reservoir, the multimammate mouse, in Africa
Source: Front Public Health. 2026 Oct 13;14:1916488. doi: 10.3389/fpubh.2026.1916488 (PMC13710268; doi:10.3389/fpubh.2026.1916488)
Supplement: Supplementary file 2 [file Table_2.DOCX]

**Table S2**. Moran’s I statistics for selected bioclimatic variables across occurrence datasets subjected to different spatial thinning distances.

| Thinning Level | N_Points | Variable | Moran’s I (observed) | Expected | SD | *p*-value |
| --- | --- | --- | --- | --- | --- | --- |
| All_data | 950 | BIO1 | 2.098397 | -0.00105 | 0.264949 | 2.22E-15 |
| All_data | 950 | BIO7 | 0.286891 | -0.00105 | 0.265102 | 0.277406 |
| All_data | 950 | BIO12 | 0.314306 | -0.00105 | 0.264436 | 0.233037 |
| All_data | 950 | BIO15 | 0.796621 | -0.00105 | 0.26513 | 0.002624 |
| 20km | 588 | BIO1 | 0.373337 | -0.0017 | 0.0054 | 0 |
| 20km | 588 | BIO7 | 0.334702 | -0.0017 | 0.005404 | 0 |
| 20km | 588 | BIO12 | 0.24105 | -0.0017 | 0.005379 | 0 |
| 20km | 588 | BIO15 | 0.346111 | -0.0017 | 0.005404 | 0 |
| 30km | 522 | BIO1 | 0.352377 | -0.00192 | 0.005388 | 0 |
| 30km | 522 | BIO7 | 0.329707 | -0.00192 | 0.00539 | 0 |
| 30km | 522 | BIO12 | 0.248019 | -0.00192 | 0.005362 | 0 |
| 30km | 522 | BIO15 | 0.316023 | -0.00192 | 0.00539 | 0 |
| 50km | 405 | BIO1 | 0.318223 | -0.00248 | 0.0058 | 0 |
| 50km | 405 | BIO7 | 0.309333 | -0.00248 | 0.005809 | 0 |
| 50km | 405 | BIO12 | 0.218213 | -0.00248 | 0.005768 | 0 |
| 50km | 405 | BIO15 | 0.284382 | -0.00248 | 0.005808 | 0 |
