# Supplementary material for: Predicted spatial distribution of the primary Lassa virus reservoir, the multimammate mouse, in Africa
Source: Front Public Health. 2026 Oct 13;14:1916488. doi: 10.3389/fpubh.2026.1916488 (PMC13710268; doi:10.3389/fpubh.2026.1916488)
Supplement: Supplementary file 3 [file Table_3.DOCX]

**Table S3**. Principal component loadings for the environmental variables retained in ecological niche models of *M. natalensis*. Values indicate the contribution of each bioclimatic variable to the first four principal components (PC1–PC4).

| **Bioclimatic Variable** | **PC1** | **PC2** | **PC3** | **PC4** |
| --- | --- | --- | --- | --- |
| BIO1 | -0.088 | -0.400 | 0.012 | -0.040 |
| BIO2 | -0.261 | 0.122 | -0.061 | -0.338 |
| BIO3 | 0.285 | -0.049 | 0.263 | 0.067 |
| BIO4 | -0.297 | 0.110 | -0.004 | -0.294 |
| BIO5 | -0.230 | -0.292 | -0.072 | -0.212 |
| BIO6 | 0.143 | -0.368 | 0.077 | 0.170 |
| BIO7 | -0.308 | 0.104 | -0.126 | -0.318 |
| BIO8 | -0.181 | -0.307 | 0.139 | -0.093 |
| BIO9 | 0.044 | -0.406 | 0.000 | 0.050 |
| BIO10 | -0.184 | -0.342 | -0.005 | -0.147 |
| BIO11 | 0.053 | -0.404 | -0.006 | 0.086 |
| BIO12 | 0.311 | -0.030 | -0.265 | -0.165 |
| BIO13 | 0.224 | -0.061 | -0.515 | -0.075 |
| BIO14 | 0.247 | -0.017 | 0.318 | -0.460 |
| BIO15 | -0.287 | -0.075 | -0.216 | 0.119 |
| BIO16 | 0.229 | -0.037 | -0.518 | -0.112 |
| BIO17 | 0.265 | -0.028 | 0.305 | -0.424 |
| BIO18 | 0.257 | 0.106 | -0.008 | 0.001 |
| BIO19 | 0.187 | -0.127 | -0.189 | -0.356 |
