# Supplementary material for: Predicted spatial distribution of the primary Lassa virus reservoir, the multimammate mouse, in Africa
Source: Front Public Health. 2026 Oct 13;14:1916488. doi: 10.3389/fpubh.2026.1916488 (PMC13710268; doi:10.3389/fpubh.2026.1916488)
Supplement: Supplementary file 4 [file Table_4.DOCX]

**Table S4.** Summary of Principal Component Analysis (PCA) of Bioclimatic Variables for *Mastomys natalensis*, Showing Standard Deviation, Variance Explained, Cumulative Variance, and Principal Component Retention.

| **PC** | **Standard deviation** | **Variance explained (%)** | **Cumulative variance (%)** |
| --- | --- | --- | --- |
| PC1 | 2.851 | 42.77 | 42.77 |
| PC2 | 2.401 | 30.34 | 73.10 |
| PC3 | 1.396 | 10.26 | 83.36 |
| PC4 | 1.056 | 5.87 | 89.23 |
